# Focus on Extracellular Vesicles: Physiological Role and Signalling Properties of Extracellular Membrane Vesicles

**DOI:** 10.3390/ijms17020171

**Published:** 2016-02-06

**Authors:** Nunzio Iraci, Tommaso Leonardi, Florian Gessler, Beatriz Vega, Stefano Pluchino

**Affiliations:** 1 Wellcome Trust-Medical Research Council Stem Cell Institute, Clifford Allbutt Building—Cambridge Biosciences Campus, Department of Clinical Neurosciences, and NIHR Biomedical Research Centre, University of Cambridge, Hills Road CB2 0PY, UK; ni238@cam.ac.uk (N.I.); tl344@cam.ac.uk (T.L.); fag21@cam.ac.uk (F.G.); bv270@cam.ac.uk (B.V.); 2 EMBL-European Bioinformatics Institute, Wellcome Trust Genome Campus, Hinxton CB10 1SD, UK

**Keywords:** extracellular vesicles, exosomes, signalling pathway, trafficking, intercellular communication, neuroscience, drug delivery, extracellular RNAs

## Abstract

Extracellular vesicles (EVs) are a heterogeneous population of secreted membrane vesicles, with distinct biogenesis routes, biophysical properties and different functions both in physiological conditions and in disease. The release of EVs is a widespread biological process, which is conserved across species. In recent years, numerous studies have demonstrated that several bioactive molecules are trafficked with(in) EVs, such as microRNAs, mRNAs, proteins and lipids. The understanding of their final impact on the biology of specific target cells remains matter of intense debate in the field. Also, EVs have attracted great interest as potential novel cell-free therapeutics. Here we describe the proposed physiological and pathological functions of EVs, with a particular focus on their molecular content. Also, we discuss the advances in the knowledge of the mechanisms regulating the secretion of EV-associated molecules and the specific pathways activated upon interaction with the target cell, highlighting the role of EVs in the context of the immune system and as mediators of the intercellular signalling in the brain.

## 1. Introduction

Extracellular vesicles (EVs) is a generic term that refers to all membrane vesicles secreted in the extracellular space [1]. As such, EVs include a broad and extremely heterogeneous population of vesicles, which posses different functions, biophysical properties and have different biogenesis routes.

Given the lack of a clear consensus on the nomenclature of EVs, the field has coined a multitude of terms to address the different types of vesicles, resulting in sub-categories that are often redundant and/or overlapping. The terms ectosomes, shedding vesicles, microvesicles and microparticle usually refer to 150–1000 nm vesicles that bud directly from the plasma membrane [2], while the term exosomes refers to smaller vesicles (30–100 nm), which are generated intracellularly by the inward budding of multivesicular bodies (MVB) and released in the extracellular space upon fusion of the MVBs with the plasma membrane [2].

The release of EVs is an extremely common and widespread biological process, which is conserved across eukaryotes, bacteria and archaea and observed in virtually every form of life [3]. In bacteria and eukaryotic microorganisms, EVs play important roles in the host-pathogen interaction and mediate the release of compounds such as virulence factors and toxins in the surrounding environment [3,4].

In animals, EVs and exosomes have been implicated in a broad, and still largely uncharacterized range of functions, such as protein clearance [5], immunity [6,7], infections [8,9,10,11], signalling [12,13,14] and cancer [15], and have been detected in a variety of biological fluids (e.g., blood, urine, saliva, amniotic fluid, malignant ascites, bronchoalveolar lavage fluid, synovial fluid and breast milk) [16]. EVs and exosomes are also being studied in connection with numerous pathological conditions and, most prominently, with neurodegenerative diseases that include Parkinson’s disease, Alzheimer’s disease, multiple sclerosis, amyotrophic lateral sclerosis, stroke and prion disease [17]. Because of their involvement in pathological processes and their presence in easy-to-access biological fluids, EVs have also attracted great interest for the potential use as disease biomarkers [17]. The field is now quickly progressing toward a more complete understanding of their range of functions *in vitro* and *in vivo* both in health and disease.

It is well established that exosomes and other classes of EVs—such as shedding microvesicles—have clearly distinct functional and morphological properties [18], and the field is now starting to develop suitable methods for their differential purification and characterization. However, a substantial amount of the literature available to date does not systematically distinguish between different vesicle populations. For these reasons, this review will focus on the physiological role and the pathological signalling of EVs in general, with a particular focus on the role of exosomes. A comprehensive introduction to EVs and exosomes, their biogenesis, structure and composition is provided by Kalra *et al.* in this focus edition [19].

### 1.1. EV and Exosome Content

In recent years numerous works have focused on providing a comprehensive characterisation of the content of EVs and exosomes, and these efforts have led to the creation of databases, such as EVpedia and Vesiclepedia [20,21], which record molecules (proteins, mRNAs, microRNAs or lipids) observed within these vesicles.

At present, Vesiclepedia [20] stores records for 92,897 proteins, 27,642 mRNAs, 4934 miRNAs and 584 lipids from 538 studies in 33 different species (database accessed on 21 September 2015). These numbers make it clear that exosomes and EVs contain an extremely broad and heterogeneous range of molecules; the following paragraphs will make an attempt at providing a description of what has been observed within vesicles and how their content changes in response to external stimuli. However, it is important to note that different studies employ a numerous different methods of vesicle isolation, sample preparation and analysis, which may influence the interpretation of the results and interfere with their comparability [22].

### 1.2. Exosomal RNAs

Exosomes and EVs have been shown to contain both short and long RNAs. EVs purified from embryonic stem cells secrete EVs enriched for mRNAs of pluripotency transcription factors (e.g., octamer-binding transcription factor 4 (Oct-4), Zinc finger protein 42 homolog (Zfp-42), Homeobox protein NANOG (Nanog), Endothelial transcription factor GATA-2 (GATA2), Homeobox protein Hox-B4 (HoxB4)), cytokines and receptors [23]. Exosomes derived from mast cell lines contain mRNAs and microRNAs (miRNAs) [24]. Additionally, these exosomal mRNAs are functional and are translated into proteins, when transferred to target cells *in vitro* [25]. This seminal work has had several implications and took the lead of subsequent work aimed at establishing the implication of extracellular RNAs in a variety of biological processes, such as the immune response, pluripotency, cancer, viral infections, angiogenesis and others [23,25,26,27,28]. Following the initial observation that exosomes traffic miRNAs [24], it was shown that exosomal miRNAs are functionally transferred to target cells, where they are able to silence target genes [29,30,31]. Exosomal miRNAs have been shown to be involved in formation of the immunological synapse [7], viral infections [30], induction of endothelial cell migration [32,33] or prometastatic inflammatory responses [34], as well as in T cell suppression [35].

In addition to mRNAs and miRNAs other RNA species have been observed within exosomes and EVs, such as viral RNAs, Y-RNAs, fragments of tRNAs, small nuclear RNA, small nucleolar RNA, piwi-interacting RNAs and long non-coding RNAs [36,37,38,39,40,41].

### 1.3. Exosomal DNA

In addition to RNA also genomic DNA has been detected in EVs. While several mechanisms for trafficking of RNA have been described (as extensively reviewed below), the incorporation of genomic DNA in EVs has not yet been completely understood. One of the proposed mechanisms suggests that fragments of genomic sequences are released into the cytoplasm during mitosis following the breakdown of the nuclear envelope, and are subsequently trafficked to specific packaging sites [42].

Genomic DNA is found in a panel of tumour cell lines such as glioblastoma, colon and gastric cancers [43]. In tumour cells, the majority of DNA associated with exosomes is double-stranded and represents a significant fraction of the genomic DNA of the cell of origin, including mutated and amplified oncogenes as well as transposable elements [26,44]. For these reasons, several lines of research are now exploring the possibility of using exosomal DNA as a circulating biomarker to identify the mutations of the parental tumour cells [44]. Besides cancer, genomic DNA has been found to be present in vesicles released from other cells; for example, fragments of genomic DNA have been identified in prostasomes, the most abundant class of EVs found in seminal fluid and originating from the epithelial cells in the prostate [45,46,47].

Despite the abundance of evidence showing the presence of DNA inside EVs and exosomes, its function still remains unclear; additional studies are needed in order to elucidate its role in physiological and pathological processes.

### 1.4. Exosomal Proteins

Recent attempts at characterizing the content of exosomes and EVs have employed techniques based on mass spectrometry to reflect the complete proteome.

This work has shown two important features of the EV/exosome proteome: (i) some proteins are present in most exosomes disregarding of the cell type of origin, while others seem to be cell-type specific; and (ii) the protein composition of exosomes does not entirely reflect the proteome of the parental cell, but rather displays an enrichment for specific proteins normally found on the plasma membrane, endosomal compartment or cytosol, while showing a depletion for other (such as nuclear) proteins [2,48]. Typical proteins often found in exosomes include tetraspanins (e.g., CD9, CD63, CD81 heat shock proteins (Hsp90), and components of the endosomal sorting complex required for transport (ESCRT) such as TSG101 and ALIX) [49,50]. However, several recent works point in the direction that exosomal proteins are probably not uniformly distributed in all the subpopulations of EVs, being enriched in some classes of vesicles while depleted in others [2,51,52]. For example, it was recently shown *in vitro* that human colon carcinoma cells secrete two distinct populations of exosomes, which are released from the apical and basolateral membranes respectively. These two populations were further shown to possess clearly distinct protein profiles; highlighting that—even in the same cell type—morphologically homogenous vesicles can have remarkably heterogeneous contents [53].

These data suggest a much greater complexity in the EV and exosome proteome than initially thought, but we are still lacking a clearly defined functional distinction between classes of EVs. The current efforts toward the development of more refined purification methods, standardized sample collection and isolation as well as improvements in protein detection techniques will help to shed additional light on the different classes and molecular composition of the vesicles that are co-purified using the current protocols [54].

### 1.5. Reactivity of Cargoes

Compelling evidence exists that cells modulate the content of exosomes and EVs in response to extracellular (extrinsic) perturbations. Stress conditions such as heat shock, hypothermia, hypoxia, oxidative stress induce remarkable changes in the protein and RNA composition of exosomes [27,55,56,57,58,59,60,61,62]. Similarly, the composition of exosomes is altered in the context of viral infections [30,63]. In addition to viruses, exosomes also traffic prion proteins, both in its normal and scrapie conformation, and mediate its propagation to other cells [11,64,65], also displaying an altered miRNA profile in the context of prion infection [37].

Thus, it is likely that the exosomal sorting machinery reacts to extrinsic cues. In support of this view, the activation of intracellular signalling pathways (e.g., mediated by cytokines) changes the exosome profile in very specific ways and can even confer to exosomes new properties and/or functions [13,14,61,66,67,68]. In line with this, we have recently shown that neural stem cells (NSCs) exposed to IFN-γ up regulate the components of the Stat1 pathway within EVs and exosomes both at the mRNA and protein level. EVs from IFN-γ-stimulated NSCs induce the activation of the Stat1 pathway in target cells via a mechanism that is mediated by the IFN-γ/Ifngr1 complex on EVs [14].

Concomitantly to the observation that the EV and exosome content is reactive to external stimuli, numerous groups have focused their efforts on characterizing the molecular machinery that allows the cells to sort specific RNAs and proteins towards exosome in a dynamic way.

## 2. Mechanisms of RNA and Protein Secretion

### 2.1. Mechanisms of miRNA Secretion

Kosaka and colleagues identified in 2010 one of the first pathways regulating the sorting of miRNAs into exosomes [69]. The authors showed that the sphingolipid ceramide is involved in the secretion of exosomal miRNAs in different tumour cell lines, and that these miRNAs retain their function when transferred to target cells. They also demonstrate that altering the levels of ceramide in the cells affects the amount of exosomal miRNAs. In particular, they show that the chemical inhibition of the neutral sphingomyelinase 2 (nSMase2)—a key enzyme involved in the biosynthesis of the ceramide—causes a decrease in the levels of exosomal miRNAs, while its overexpression induces an increase in the secreted amounts of miRNAs. Interestingly, the knockdown of members of ESCRT did not interfere with the secretion of miRNAs, thus suggesting that the two phenomena are independent [69]. However the exact molecular determinants regulating the sorting of miRNAs to exosomes remained elusive.

Most of the later studies about the mechanism of RNA secretion also focused on miRNAs. Exosomal miRNA profile in exosomes was shown to be the result of a tightly regulated process that does not simply reflect the transcriptional landscape of the parental cell [24,30,70,71,72]. In 2007, it was shown that a 6-nt sequence address different miRNAs in the nucleus through a mechanism yet to be defined [63]. Thus, a similar mechanism might also direct the secretion through specific interaction between a *mobile motif* in the sequence of miRNAs and carrier proteins.

The group of Sanchez-Madrid demonstrated that such a motif is present in miRNAs and controls their localization into primary T lymphoblast-derived exosomes [70]. First, they were able to identify a populations of miRNAs preferentially sorted into exosomes, and then—through multiple alignments of the mature sequences—they characterized the motif specifically enriched in these secreted miRNAs as the 4 nt GGAG. They proved by mutagenesis that this motif is able to modulate directly the miRNAs loaded into exosomes. In the same study the authors also identified for the first time the heterogeneous nuclear ribonucleoprotein A2B1 (hnRNPA2B1) as the carrier responsible for miRNA secretion in exosomes [70]. HnRNPA2B1 is a RNA-binding protein, expressed ubiquitously, and involved in the subcellular localization of specific mRNAs in neurons [73], with a role also in the context of HIV infection [74]. Considering the similarities between viral production and exosome biogenesis, it is not completely surprising that these two phenomena have a similar RNA sorting mechanism [75]. Indeed hnRNPA2B1 was shown to bind specifically the exosomal miRNAs containing the GGAG motif and thus to control their sorting into exosomes [70]. Moreover, the authors demonstrated that the sumoylation of hnRNPA2B1 promotes its localization in the exosomes and, more importantly, the interaction with the GGAG motif in the exosomal miRNAs. These findings raise the possibility that post-translational modifications of proteins may play a key role in their loading into exosomes. The great potential of such a mobile motif for the artificial loading of selected miRNAs in vesicles and, in the longer term, for the design of functionalized exosomes as a novel therapeutic approach.

Whether the primary function of a carrier such as hnRNPA2B1 is (i) to mediate the secretion of a specific subset of miRNAs for cell-to-cell communication; or (ii) to eliminate undesired miRNAs from the cell, remains to be further clarified.

More recent studies have added novel levels of complexity to the mechanism that guides the secretion of miRNAs into exosomes. Interaction with target mRNAs, nontemplated nucleotide additions as well as new biogenesis of miRNAs inside the exosomes, have been demonstrated to be involved in the exosomal miRNA loading [76,77,78].

Squadrito and colleagues employed bone marrow-derived macrophages (BMDMs) as a cellular model to further investigate the mechanism of exosomal miRNA sorting. In their studies, the activation state of the BMDMs contributes to the regulation of miRNA secretion in exosomes. The stimulation of BMDM with interleukin (IL)-4 leads to the enrichment of a subset of miRNAs in exosomes *vs.* cells. The transcriptional changes observed after IL-4 treatment modify the pool of the miRNA target sequences (*i.e.*, mRNAs), thus controlling miRNA sorting to exosomes [77]. The miRNAs whose target mRNAs are decreased in IL-4-treated BMDM are specifically enriched in exosomes, without affecting the total amount of cellular miRNAs. This phenomenon is mediated by a redistribution of miRNAs from the P bodies (where the cellular miRNAs are active), to the MVB (where they are sorted for exosome-mediated secretion) [77]. Interestingly these findings seem to support the idea that exosomal miRNA secretion is a *cleaning* mechanism used by cells to maintain the miRNA:mRNA homeostasis.

Koppers-Lalic and colleagues have identified another possible mechanism of exosomal miRNA sorting in human B cells. In their study the distribution of extracellular miRNAs is partly regulated by nucleotidyl transferase-mediated posttranscriptional modifications, known as nontemplated nucleotide terminal additions (NTAs) [76]. Exosomal miRNAs are enriched in 3′ end uridylated isoforms, while miRNAs retained in cells are preferentially adenylated at the 3′ end, and the more adenines are added, the more the miRNAs are retained. These findings were validated in naturally occurring EVs from human urine samples, thus confirming that the addition of nucleotides at the 3′ end of miRNAs could impact their distribution in exosomes [76]. This work opens new questions about the role of different pathways within the cells (e.g., RISC loading complex, carrier proteins, RNA editing enzymes) and how they interact to finally promote the secretion of specific miRNAs via exosomes.

Melo and colleagues have finally described a completely new mechanism of cell-independent maturation of miRNAs within breast cancer-derived exosomes, thus adding a further level of complexity to the whole phenomenon of exosomal miRNA secretion. First, they showed that miRNAs are generally enriched in exosomes derived form cancer *vs.* normal cells. Then, they proved that the miRNA biogenesis is active in exosomes, as a decrease of pre-miRNA levels over time is paralleled by a proportional increase of the mature sequences. Notably, several components of the miRNA biogenesis machinery (*i.e.*, Dicer, TAR RNA-binding protein 2 (TRBP) and Argonaute (AGO) 2) were found functional within cancer exosomes, while not detectable in those from normal cells [71]. Dicer was specifically proved as the key component responsible for the oncogenic potential of cancer exosomes when injected together with nontumorigenic human mammary epithelial cells into the mammary fat pads of female nude mice [72]. Also, the authors validated their findings with serum exosomes purified from cancer patients, showing that (i) these vesicles contain active for of the endoribonuclease Dicer; and (ii) Dicer is necessary to induce tumour formation using the same experimental approach in nude mice [72]. Interestingly, the authors showed the interaction between Dicer and the transmembrane anchor protein sialophorin (or CD43), thus suggesting a role for CD43 in the transport of Dicer and possibly of miRNAs into exosomes.

### 2.2. Mechanisms of mRNA Secretion

In addition to miRNAs, also mRNAs and proteins where found to be selectively enriched/depleted in EVs compared to donor cells.

Batagov and colleagues found that exosomes-enriched RNAs tend to have a shorter half-life than cellular RNAs. They also found 1458 bp linear motifs that correlate with the RNA secretion fraction (*i.e.*, the ratio of exosomal *vs.* cellular RNAs) [79]. Although none of these motifs is individually found in more than 24% of exosomal RNAs, this result suggests that the combination of multiple motifs or conserved secondary structures shared by these motifs might be responsible for shuttling RNAs towards exosomes. Bolukbasi *et al.* identified a 25 nt stem-loop forming sequence in the 3′-UTR of twenty EV-enriched RNAs [78]. They also show that this stem-loop contains a binding site for miR-1289, and that overexpression of the miRNA increases the enrichment in EVs of RNAs from a vector carrying the stem-loop in the 3′-UTR [78].

More recently, Szostak and colleagues found that none of the RNA sorting motifs previously identified is enriched in EVs from the murine hepatic cell line MLP-29, while they show that a novel 12 nt motif part of a stem-loop region is present in ~40% of EV-enriched mRNAs. Moreover, they also show that cloning the motif in a luciferase reporter induces the secretion of the reporter mRNA in EVs [80].

All these studies—although discordant—point in the direction that there exist a mechanism that recognises short RNA motifs, either linear or structural, and mediates RNA export towards EVs/exosomes. The fact that none of these studies agrees on the sequence identified suggests that the RNA sorting machinery might rely on different players in different species and/or cellular systems.

Further experiments are required to obtain a comprehensive understanding of the molecular mechanism by which the RNA sorting machinery operates.

### 2.3. Mechanisms of Protein Secretion

In addition to miRNAs and mRNAs, proteins also seem to be selectively sorted into EVs, and also in this case the mechanisms behind this process are still largely uncharacterised.

The current opinion is that there are at least three independent mechanisms responsible for the sorting of exosomal proteins: lipid-mediated, tetraspanins-mediated and ESCRT-mediated [81]. It seems that these mechanisms coexist and are responsible for the sorting of different proteins and/or for the loading of different subpopulations of vesicles. Multiple components of the ESCRT complex posses ubiquitin-binding activity, suggesting that protein ubiquitination might be a posttranslational modifications involved in the sorting of exosomal proteins [82,83]. In support of this hypothesis, it was shown that the ubiquitin-protein ligase NEDD4 ubiquitinates HTLV-1 Gag protein and mediates its recruitment by Tsg101, which in turn causes its sorting toward MVBs [84]. Similarly, it was recently found that the sumoylation of hnRNPA2B1 is a post-translational modification that targets the protein bound to miRNAs towards exosomes, thus suggesting that sumoylation might play a more general role in sorting protein towards exosomes [70]. In addition to ESCRT, tetraspanins have been involved both in the formation of exosomes and in the sorting of exosomal proteins. Tetraspanins are a family of integral membrane proteins with four transmembrane domains typically enriched in exosomes. For example, the tetraspanin CD63 binds to the LMP1 protein of the Epstein Barr Virus and mediates its sorting in MVBs [85]. Moreover, Shen *et al.* have shown that for a variety of proteins, a higher order oligomerization and the capacity to bind the plasma membrane are sufficient to induce the sorting toward MVBs [86].

In addition to ESCRT and membrane proteins, also lipids have been implicated in exosome secretion and sorting of cargo proteins [81]. For example, enzymes that metabolise sphingolipids were shown to modulate exosome secretion by neuron and—as a consequence—modulate the clearance of Amyloid-β-peptide by microglia [87].

These and other data support the hypothesis of lipid-regulated protein trafficking, but it is still unclear whether they modify vesicle content by modulating the formation and secretion of specific classes of vesicles or whether they also directly mediate the incorporation of specific proteins in exosomes.

## 3. Role of EVs and Exosomes in Physiology

The earliest studies on EV functions date back to the early 1980s, when they were characterized as *exfoliated vesicles from various normal and neoplastic cell lines with 5′-nucleotidase activity* [88]. In 1982, Stegmayr and Ronquist published one the first studies demonstrating the functional interaction between prostate cell-derived EVs—called prostasomes—and sperm cells, responsible for the promotion of sperm progressive motility [89]. In 1983 the secretion of exosomes was first described in two independent studies as a part of the reticulocyte maturation, acting to remove obsolete membranes and proteins in a process of “*reverse endocytosis*”—thus called exosomes [90,91]. In recent years, several works have shown that a variety of cell types are capable of releasing EVs/exosomes in the extracellular space both *in vivo* and *in vitro*.

Most of the studies regarding the possible physiological roles of EVs/exosomes have been based on indirect *in vitro* evidences, especially in the context of the immune system and cell-to-cell communication [92]. Nevertheless the definitive evidence for their physiological role remains elusive.

### 3.1. Exosome Function and the Immune Response

In 1996, a pioneering study by Raposo and colleagues demonstrated that exosomes derived from both human and mouse B-lymphocytes spread antigens bound to the class II major histocompatibility complex (MHC-II). Notably, these vesicle-associated complexes were capable of activating MHC class II-restricted T cell responses, suggesting a role for exosomes in antigen presentation *in vivo* [93]. Furthermore, B cell-derived exosomes specifically interacted with the membrane of follicular dendritic cells (DCs) derived from human tonsils, further supporting their active secretion *in vivo* [94,95].

Exosomes from DCs were shown to bear peptide-MHC-I, -II and other T-cell costimulatory molecules, such as CD80/B7.1 and CD86/B7.2. As for B cells, these exosomes are able to induce an immune response by spreading MHC-antigen complexes to other DCs and also to both CD4^+^ and CD8^+^ T-lymphocytes, thus mediating their activation [6,96,97,98,99,100,101,102,103]. Moreover, adhesion molecules associated with vesicles can contribute to this phenomenon: in fact exosomes secreted by mature DCs expose intercellular adhesion molecule (ICAM)-1, which is able to interact with lymphocyte function-associated antigen (LFA)-1 expressed by CD8^+^ DCs and T-lymphocytes [104,105].

In addition to transmembrane proteins, other functional molecules were identified in immune cell-derived exosomes. Montecalvo and colleagues demonstrated that different subsets of miRNAs are exchanged between DCs through exosomes, depending on the DC maturation stage. Notably the authors showed that the exosomal miRNAs maintain their ability to repress target sequences in the recipient cells, and that the predicted targets are crucial mRNAs for the biology of the DCs, involved in the differentiation, cytokine synthesis and transforming growth factor (TGF)-β signalling [29]. Mittelbrunn and colleagues have demonstrated that the exosomal miRNAs are shuttled in a unidirectional way from T cells to antigen presenting cells (APCs) in the microenvironment of the immune synapse (IS) [7]. The ISs are highly specialized cell-to-cell contact sites crucial for antigen presentation and the regulation of T-cell activation [106]. In this context the antigen binding induces the formation of the IS, with the resulting polarization of the T-cell MVB towards the APC. As a consequence, the secretion of exosomes is increased and, ultimately, the antigen-driven IS formation promotes the delivery of exosomal miRNAs to APCs. Moreover, only exosomes transferred in the context of the IS appear to be functional in the target APCs. As a proof of concept, the T cell-derived exosomal miR-335 was shown to target Sox4 mRNA in recipient APCs, supporting the possibility that exosomal miRNAs may play a role in the regulation of the immune response [7].

Bone marrow-derived macrophages (BMDMs) infected with *Mycobacterium tuberculosis* (Mtb)—or other related mycobacteria—secrete exosomes [107,108] that contain Mtb-derived glycopeptidolipids (GPLs). Exosome-associated GPLs promote the secretion of tumour necrosis factor alpha (TNF-α) and chemokine (C-C motif) ligand 5 (CCL5/RANTES)—two key mediators of the innate inflammatory activation—in recipient uninfected macrophages. This pro-inflammatory response is dependent on the interaction with the Toll-like receptors (both TLR-2 and -4) and its associated adaptor molecule myeloid differentiation primary response gene 88 (MyD88) in the target macrophages. Mitogen-activated protein kinase (MAPK) and the nuclear factor kappa-light-chain-enhancer of activated B cells (NF-κB) are the key pathways involved in this phenomenon [108]. Notably these infected BMDM-derived exosomes are able to induce the production of TNF-α and IL-12 *in vivo*, when injected intranasally into mice. The secretion of this cytokines was proved to be functional, as both neutrophils and macrophages were recruited in the lungs of these mice [107].

BMDM-derived exosomes are also capable to deliver functional IL-1β—another key mediator of the innate pro-inflammatory response—in mice. Qu and colleagues identified in extracellular ATP the trigger for the secretion of exosome-associated IL-1β, and established the role of the P2X purinoceptor 7 (P2X7R) in mediating the effect of the ATP [109]. Ramachandra and colleagues have identified another mechanism of immune response activation mediated by exosomes from Mtb-infected BMDM. As described for B cells and DCs, Mtb-infected BMDM exosomes can activate target T lymphocytes by transferring peptide–MHC-II complexes, in a process that is ATP-dependent [110]. Taken together these studies support an active role for macrophage-derived exosomes in the antimicrobial immune surveillance.

EVs can also mediate (i) immunosuppressive effects on T lymphocytes and natural killer (NK) cells; and (ii) the induction of T regulatory and myeloid cells to further inhibit the immune response [63,111,112,113,114,115,116,117,118,119,120,121].

Placenta-derived exosomes purified from the blood of pregnant women carry immunosuppressive molecules that induce tolerance toward the foetus. Exosomes from pregnancies delivering at term bear higher levels of these immunosuppressive molecules compared with exosomes from preterm birth. In this study, Fas ligand (FasL) was identified as the putative agent responsible for the inhibition of T-cell surface glycoprotein CD3 zeta chain (CD3- ξ) and tyrosine-protein Janus kinase 3 (JAK3), ultimately suppressing the activity of maternal cytotoxic T lymphocytes and NK cells [10]. A few years later Hedlund and colleagues showed a similar effect mediated by exosome-associated UL16 binding proteins (ULBPs), a novel family of ligands of the activating NK cell receptor NKG2D in human. Placental exosomes bear ULBP1–5 and induce down-regulation of the NKG2D receptor on NK cells, CD8^+^ and γδ T lymphocytes, thus inhibiting their cytotoxicity activity *in vitro* [122].

EVs are also stimulatory for the immune system, with the final effect depending on many aspects, such as the identity of the donor and target cells, as well as the biological context in which this interaction takes place. This dichotomy appears also in the epithelia, another microenvironment where the EV-mediated signalling with the immune system was described [123,124,125].

In 2001 van Niel and colleagues employed human intestinal epithelial lines to demonstrate that these cells secrete exosomes exposing accessory molecules such as MHC-I and -II that may be involved in antigen presentation, a mechanism already proved for the DCs. They showed that the secretion of exosomes is further increased after IFN-γ treatment, suggesting the relevance of this phenomenon in inflammatory conditions [123].

Two years later, the same group confirmed *in vivo* the immunogenicity of the peptide-MHC-II associated with exosomes, showing the migration of these complexes towards the gut lymph nodes with a pro-inflammatory effect in mouse [124]. Prado and colleagues showed that exosomes derived from the bronchoalveolar fluid of allergen-tolerized mice are capable to induce in turn tolerization when injected intranasally into recipient mice. After exosome administration, the authors demonstrated the inhibition of both specific IgE and IgG1 antibodies in the treated mice, as well as a decrease of Th2-like cytokines, with a parallel increase of TGF-β. The final outcome of these experiments proved that exosomes derived from a tolerogenic context are capable to reduce allergen-induced airway inflammation, inducing also a stable protection against allergic sensitization *in vivo* [126].

An additional function of exosomes that was recently described is related to their role in the context of viral infections. As such, de Carvalho *et al.*, showed that exosomes from CD4^+^ T cells inhibit HIV-1 infection *in vitro*, suggesting that exosomes might act as decoy receptors for the virus, binding its surface proteins and preventing its interaction with target cells [127].

### 3.2. Exosome Function in the CNS

In the context of immune response regulation, the intercellular signalling mediated by EVs and exosomes appears to be relevant also for the cells of the central nervous system (CNS).

Microglial cells are the resident macrophages in the brain, responsible for the first intrinsic immune response and tissue repair [128,129]. The role of microglia remains a matter of debate, as both bright and dark sides in its function have been highlighted, both during brain development and disease [130,131,132,133].

After CNS injury, microglial cells quickly increase the expression levels of MHC-I and -II molecules, playing a prominent role as antigen presenting cells. During brain inflammation, T lymphocytes cross the blood–brain barrier (BBB) and directly interact with microglia to recognise antigens and ultimately to mediate a pro-inflammatory response [134]. In this context microglia were shown to secrete exosomes containing proteins already reported in vesicles from B lymphocytes and DCs (e.g., antigen-MHC complexes) [135]. The treatment with astrocyte-derived ATP induces in mouse microglia the shedding of EVs, which bear functional IL-1β [136], a feature that has also been confirmed in EVs from mouse astrocytes [137]. The ATP binding to the P2X7R activates the acid sphingomyelinase, which plays a crucial role for the subsequent induction of EV shedding and secretion of EV-associated IL-1β via a MAPK-dependent mechanism [138]. Verderio and colleagues demonstrated the presence of microglia-derived EVs in the cerebrospinal fluid (CSF) of mice with CNS inflammation, thus suggesting a possible role for circulating EVs as biomarkers of diseases [139].

Oligodendrocytes (ODCs) secrete exosomes enriched in crucial components of the myelin sheaths—such as the myelin proteolipid protein (PLP), myelin basic protein (MBP) and myelin oligodendrocyte glycoprotein (MOG)—together with several proteins with proposed functions in the trophic support of axons. Furthermore, the intracellular levels of calcium regulate the secretion of these exosomes, suggesting that the whole process is strictly regulated to serve a protective role for neurons [140].

The interplay between ODCs and neurons seems to be even more articulated, as the latter are capable to modulate the release of exosomes by ODCs through the neurotransmitter glutamate [139]. In this study, the electrical activity of axons induces the release of glutamate, thus evoking in turn ODC Ca^2+^ signals through *N*-methyl-d-aspartate (NMDA) and α-Amino-3-hydroxy-5-methyl-4-isoxazolepropionic acid (AMPA) receptors. Ultimately this pathway triggers the release of ODC exosomes containing specific protein and RNA cargoes that are internalized by neurons. Notably the authors demonstrate that the ODC-derived exosomes protect target neurons from oxidative stress and starvation [139]. ODCs secrete exosomes with auto inhibitory effects on their own growth. Nevertheless conditioned neuronal medium appears to be able to decrease the production of such exosomes, thus controlling the terminal differentiation of ODCs and finally biogenesis of myelin sheaths [140].

Similarly to what happens with ODCs in the CNS, also Schwann cells (SCs) in the peripheral nervous system (PNS) can exchange exosomes with neurons. In 2013 Lopez-Verrilli and colleagues showed that SC-derived exosomes are selectively transferred to the dorsal root ganglia axons, enhancing regeneration in injury models both *in vitro* and *in vivo* [141].

Other multiple interactions were suggested in the context of the brain, further increasing the level of complexity of this EV-mediated cell-to-cell communication. ODC-derived exosomes can be internalized also by microglia via macropinocytosis, in a phenomenon possibly relevant in the context of ODC membrane clearance [142]. Moreover, EVs secreted by microglia affect in a dose-dependent way the activity of neurons, mainly at the presynaptic site. In fact, Antonucci and colleagues demonstrated that the microglia-derived EVs are capable to increase the neuronal sphingolipid metabolism—via the action of the acid sphingomyelinase - thus stimulating the synaptic activity [143].

As for ODCs and microglia, also astrocyte-derived exosomes were proved to be important regulators of neuronal biology. Synapsin I—an oligomannose-binding glycoprotein—was identified by Wang and colleagues as the crucial astrocyte-derive exosome-associated protein capable of (i) promoting neurite outgrowth from hippocampal neurons and (ii) inducing survival of cortical neurons upon oxidative stress [144].

Exosomes purified from primary cultures of cortical neurons contain both vascular endothelial growth factor (VEGF) and fibroblast growth factor (FGF)-2, thus suggesting a possible role in the communication with endothelial cells to promote angiogenesis [145]. Another independent study confirmed that the release of EVs is a tightly regulated process in neurons, and that it is driven by the glutamatergic synaptic activity [146]. Furthermore, Korkut and colleagues showed that the morphogens Wnt proteins are secreted on exosomes both during Drosophila development (at the larval neuromuscular synapses) and in human cells [147]. Importantly, these findings suggest a broader role for the EV-mediated intercellular signalling: for example during development, many studies demonstrated that components of Wnt, Notch and Hedgehog pathways use extracellular vesicles to signal with the microenvironment [148,149,150]. Also in the context of cancer the transfer of pathway activation via EVs has been described for many factors such as MET [151], TGF- β [152], Wnt [153], BCR [154] and NOTCH3/STAT1 [155]. For further information on the signalling properties of EVs in the tumour environment, the reader is referred to the specific review in this focus edition [156].

Despite the increasing evidences about the functions of vesicle transfer an objective limitation common to the vast majority of these studies lies in the step of purification and concentration *in vitro*. Moreover, considering that almost all cell types secrete EVs and exosomes, it is important to remember that the *in vivo* situation in the EV-mediated intercellular signalling is much more complex as likely multidirectional and strongly depending on the specific context.

## 4. Role of EVs and Exosomes in Pathology

EVs have been tightly linked to tumorigenesis [157], spread of viruses and pathogenic agents such as HIV-1 [158], as well as being implicated in the propagation of protein aggregate disorders. It is becoming increasingly evident that the most prominent and well-studied neurodegenerative diseases share common cellular and molecular pathological mechanisms, among which protein aggregation and inclusion body formation play an important role [159]. These aggregates include β-amyloid (Aβ) in senile plaques and tau in neurofibrillary tangles (NFTs) in Alzheimer’s disease; α-synuclein in Lewy bodies and Lewy neurites in Parkinson’s disease; TAR-DNA-binding protein 43 (TDP-43) and superoxide dismutase (SOD1) aggregates in ALS; polyglutamine (polyQ)-rich huntingtin inclusions in Huntington’s disease and prion plaques in Creutzfeldt-Jakob disease (CJD).

Aβ-derived peptides [160], α-synuclein [161], and the abnormal pathogenic cell surface prion protein PrP^Sc^ [162] have all been found associated with EVs. For these reasons, exosomes have recently been proposed as the Trojan horses of neurodegeneration [163]. However, EVs and their components represent at the same time a novel class of potential therapeutic targets, and a potential therapeutic tool for tissue regeneration and immune response modulation.

In the following sections we describe the current state of knowledge regarding the relations between EVs and neurodegenerative diseases, the studies done to establish these correlations, and the potential relevance of EVs as therapeutic agents.

### 4.1. Prions

Several studies have explored EVs in the context of the interneuronal spreading of transmissible prion disorders such as CJD, Gerstmann-Sträussler-Scheinker disease, fatal insomnia and kuru in humans and bovine spongiform encephalitis (BSE) and scrapie in bovines and sheep respectively. The aetiological agent of these diseases is the misfolded form of the prion protein (PrP). This protein, in its native state (PrP^C^), is expressed in all tissues of the human body, with the highest levels of expression observed in the CNS [164]. PrP^Sc^, the misfolded form of PrP^C^, is infectious and it acts as a seed to catalyse the conversion of PrP^C^ into PrP^Sc^. PrP^Sc^ forms aggregates that give rise to amyloid fibres, which in turn alter the function of the nervous system and lead to the manifestation of the disease [165].

Prion disease is most commonly of infectious origin: it starts in the gut with the ingestion of contaminated food, and then spreads to lymphoid organs from where it can reach the peripheral nervous system and, ultimately, the CNS [166]. It has been observed that PrP^Sc^ can hijack tunnelling nanotubes to spread from one cell to another [167]. However, this mechanism of communication can traverse only short distances; a potential parallel mechanism involving exosomes might facilitate PrP^Sc^ transfer over large distances, thus promoting the peripheral spread of prions [168]. In this scenario, EV-mediated spreading of PrP^Sc^ would either be mediated by direct EV diffusion with the blood stream or by means of EV internalization inside blood cells and their subsequent interaction with target cells throughout the organism. In fact, both PrP^C^ and its pathological form PrP^Sc^ have been isolated in association with exosomes, and PrP^Sc^-containing exosomes were shown to be infectious in both animal and cell bioassays [11].

Ultrastructural studies confirmed the presence of both PrP^C^ and PrP^Sc^ in MVB, as well as in the late endosome, confirming the involvement of the exosomal pathway [169,170]. Furthermore, it has been reported that PrP^Sc^ and PrP^C^ are secreted in exosomes from a PrP-expressing neuronal cell line, and exosomes can spread PrP^Sc^ in uninfected recipient [162].

Additionally, Fevrier and colleagues have also shown that the inoculation of ovine PrP^Sc^-positive EVs in the brain of transgenic mice that express ovine PrP induced acute neurological symptoms that eventually led to the death of all animals [11].

Summarizing, there is strong evidence that indicates that the cell-to-cell spread of the prion disease is mediated, at least in part, by the EV or exosome-mediated transport of PrP^Sc^ from infected to uninfected cells.

### 4.2. Parkinson’s

The protein α-synuclein is a mediator of neurodegeneration in PD and its aggregation plays a central role in the pathology. PD is characterized by intracellular aggregates known as Lewy bodies [164,168,169], comprised primarily of α-synuclein, but with other proteins and lipids also present [168].

It was recently observed that α-synuclein can be (i) transferred intercellularly [164,168,169]; and (ii) detected in the plasma and CSF of patients as well as in the culture supernatant of neuronal cells. Specifically, it was also shown that α-synuclein can be transferred from neurons to astrocytes, which in response induce the expression inflammatory genes [171].

Following these observations, various groups have now started to investigate the various possible mechanisms of cell-to-cell α-synuclein transfer. In fact, recent data show that α-synuclein secretion might be mediated by tunnelling nanotubes [172] or direct secretion of the protein [173]. Additionally, Emmanouilidou and colleagues have shown that α-synuclein is also secreted in a calcium responsive manner within EVs that possess the typical characteristics of exosomes [155].

The mechanisms by which extracellular α-synuclein seeds oligomerization and confers toxicity is not well elucidated. Details regarding the specifics of interactions between α-synuclein and exosomes require further study, and the role and extent that exosomes play in PD pathogenesis is still unclear.

### 4.3. Alzheimer’s

AD is the most common form of age-associated dementia in humans [171]. AD is characterized by the accumulation of extracellular Aβ plaques and intracellular neurofibrillary tangles of hyper phosphorylated tau protein that have neurotoxic effects and, as a consequence, lead to a slow and progressive loss of neurons. The Aβ peptide is formed by the consecutive proteolytic cleavage of the transmembrane amyloid precursor protein (APP) by β-secretase and γ-secretase [172]. Under physiological conditions, tau protein is a microtubule-stabilizing protein that is predominantly expressed in neurons and regulates axonal transport [173], but tau’s hyper phosphorylation leads to its dissociation from microtubules and aggregation into insoluble fibres.

In the context of AD pathogenesis, exosomes were initially studied in connection with the formation and transport of the Aβ peptides to the extracellular environment.

For example, Rajendran and colleagues showed in 2006 that MVBs are the cellular compartment where β secretase cleaves APP into Aβ, which in turn is secreted within exosomes upon fusion of the MVB with the plasma membrane [174]. These data suggest a role for exosomes in AD pathogenesis, a view that is also supported by the observation that exosomal protein accumulate in the plaques of AD patient brains. Additionally, exosomal proteins were also found to accumulate in the plaques of AD patient brains, suggesting a role in the pathogenesis of AD [174].

Similarly, exosomes were also shown to be involved in the spreading of tau-mediated toxicity [175]. For example, a recent study showed that tau fibrils are readily and spontaneously taken up by cells *in vitro*, and even small quantities of tau fibrils induce the formation of large amounts of filamentous neurofibrillary tangles in tau-expressing cells [176]. In addition, it was also shown that tau filaments can spread *in vivo* upon injection of mutant tau protein in the brain of wild-type mice [177].

Further studies demonstrated that dimeric and trimeric tau species are found in exosomes secreted by tauopathy cellular models as well as in the CSF of AD patients [175], thus suggesting that exosomes might transport oligomeric tau species that serve as a seed to induce fibre formation in other cells. Additional work is still required to clarify whether oligomerization takes place in the exosomes or whether oligomers are directly packaged into them. The localization of tau inside vesicles would point towards an active packaging pathway rather than nonspecific secretion via extravesicular membrane-bound tau. Nevertheless, either route would facilitate the delivery of tau into neurons and contribute to the spread of disease.

Thus, a thorough elucidation of the mechanism by which tau is spread can be integral to efficient targeting of tau in a therapeutic context.

### 4.4. Amyotrophic Lateral Sclerosis

In ALS, aggregates of misfolded and mutated copper zinc superoxide dismutase (SOD)1 disseminate in a spatiotemporal manner connecting upper and lower motor neurons [178].

In a recent study Haidet-Phillips *et al.* have shown that progenitor cell-derived astrocytes differentiated from post-mortem ALS brains selectively kill motor neurons in a co-culture system [179]. Moreover, they also demonstrated that knockdown of SOD1 by short hairpin RNAs (shRNAs) in astrocytes abrogates their toxicity on motor neuron in the co-culture system. Finally, by incubating motor neurons with astrocyte-conditioned medium, they proved that the toxicity is mediated by secreted factors. In a recent paper Grad *et al.*, showed that SOD1 misfolding can be propagated from cell to cell via exosome-dependent mechanism [180].

These data, together with the evidence that SOD1 is secreted in exosomes [181], suggest that SOD1-mediated toxicity might be spread by exosomes-mediated cell-to-cell transfer.

### 4.5. Multiple Sclerosis

Multiple sclerosis (MS) is an inflammatory disease of the CNS in which perivascular infiltration of self-reactive T lymphocytes leads to demyelination (both primary and secondary), and axonal damage. The pathophysiology of MS is the result of a complex interplay between numerous players, among which autoreactive T lymphocytes, monocytes, microglia and endothelial cells [182].

In this context, EVs may play multiple roles [183], as various cell types secrete or modulate the content of EVs in response to the inflammatory environment. For example, upon activation of the BBB, endothelial cells release EVs, which can be readily detected in the plasma of MS patients during disease relapses [184].

The evidence about the effects of EVs on the microenvironment is still sparse, but there are reports suggesting that astrocyte-derived EVs contain metalloproteinases, which contribute to the BBB disruption [185], therefore facilitating the trans-endothelial migration of inflammatory cells in the CNS. Consistently with this finding it was shown that endothelial EVs derived from the plasma of MS patients can bind and activate monocytes and induce their trans-endothelial migration *in vitro* [186]. Subsequently, a different study confirmed these results and further showed that only the plasma from relapsing MS patients—while not that of remitting patients or controls—induced monocytes transendothelial migration in a brain microvascular endothelial cells (BMVEC) model [187].

Recently, a study on circulating RNAs identified 7 miRNAs significantly deregulated in the plasma of MS patients compared to healthy age and gender-matched controls (miR-614, miR-572, miR-648, miR-1826, miR-422a, miR-22 and miR-1979) [188]. Although this study does not directly address whether these circulating miRNAs are released in EVs, it suggests that miRNA secretion might play a previously underestimated role in MS pathophysiology.

A few recent studies have investigated the role of EVs in experimental autoimmune encephalomyelitis (EAE), an animal model of MS. For example, Verderio *et al.* recently showed that the administration of myeloid EVs in the brain of EAE mice induces the infiltration of CD45^+^ inflammatory cells and Iba1^+^ microglia [138]. Despite these findings that suggest a detrimental, pro-inflammatory function for EVs in MS, other reports attribute them an immune suppressive, modulatory role.

In fact, Gatson *et al.* compared exosomes in late pregnant and virgin EAE mice finding that exosomes derived from the serum of pregnant mice suppress T cell activation *in vitro* [189]. Similarly, it was also shown that exosomes from the serum of pregnant EAE mice induce the maturation of oligodendrocyte precursor cells (OPC) and facilitate their migration to CNS lesions [190]. These findings suggest that during pregnancy serum exosomes might have protective immune modulatory effects that contribute to the reduction of relapses in EAE during gestation. More recently, another study has shown that exosomes derived from DCs expressing membrane-associated TGF-β inhibit the progression of EAE and decrease Th1 and IL-17 responses [191].

Ultimately, EVs appear to have paradoxical functions in MS (and models thereof): while in specific cases they may promote the repair of demyelinating lesions, their immune-modulatory nature contributes to the disruption of the blood-brain barrier and subsequent spread of inflammation in the brain parenchyma.

### 4.6. Stroke

Stroke is the second leading cause of death in developed countries, with a mortality rate higher than cancer [192] and being the most common cause for disability in adults. In ischemic stroke, the severity of the disease is dependent on the reduction of cerebral blood flow [193]. Between an ischemic core and regularly perfused tissue lies an area with functionally inactive, yet viable neurons named penumbra. Current treatment regimens for the acute phase of the disease target the penumbra to decrease the amount of cell death. In addition to classical reperfusion strategies [194], current targets for neuroprotection include reduction of inflammation, oxidative stress, excitotoxicity, apoptosis and cell-based therapies [195].

Recent evidence suggests that EVs play an important role in the pathogenesis of stroke. In fact, calcifying EVs have been found to be involved in collagen calcification. Calcifying EVs, also named *matrix vesicles*, have historically been described in bone development [196,197]. Moreover, calcifying EVs derived from macrophages and smooth muscle cells might also contribute to vascular calcification [198]. In this context, EVs serve as foci for the formation of microcalcifications, which lead to plaque instability, rupture and subsequently, development of stroke [199].

Additionally, there is also evidence suggesting a role for endothelial and platelet-derived EVs in ischemic stroke. Endothelial activation—and consequently platelet activation—plays a major role in the pathophysiology of stroke: endothelial activation promotes the activation and adhesion of platelets, thus contributing to thrombus formation. Moreover, the activated endothelium expresses adhesion molecules (such as E- and P-selectins) that promote the recruitment and extravasation of leukocytes, which in turn migrate to the injured tissues and contribute to reperfusion damage [200,201].

In this context, multiple studies have described that increased levels of EVs released by activated endothelial (as determined by the expression of the E-Selectin CD62E) are correlated with recent ischemic episodes and associated with indices of neurological damage, suggesting that EVs of endothelial origin play a role in cerebrovascular disease [202,203].

## 5. EVs/Exosomes as Therapeutics

The recent advances in stem cell biology have raised great expectations that diseases and injuries of the CNS may be ameliorated by the development and delivery of non-hematopoietic stem cell-based therapeutics. Most of the more classical experimental cell therapies-based on the focal injection of neural lineage-committed progenitor cells—have in fact failed to foster substantial tissue repair in disease models where the anatomical and functional damage is widespread and an inflamed and/or degenerative microenvironment co-exists [204]. On the other hand, solid evidence has been accumulated from many laboratories and in many conditions that NSCs survive transplantation procedures within the host CNS, specifically migrate within the damaged tissue, and protect the nervous system from inflammatory and other forms of damage. Irrespectively from the experimental disease course, the neuropathological features and type of inflammation, the overall functional recovery obtained following NSC transplantation is usually poorly correlated with the absolute numbers of the donor-derived progeny *in vivo* in [204]. This has been shown for NSC transplants in experimental Parkinson’s or Huntington’s diseases, where NSCs very rarely differentiate into neurons *in vivo* while mediating a significant clinico-pathological recovery. Similarly, mice with acute cerebral ischemic stroke or intracerebral haemorrhage improve at both behavioural and pathological levels, in spite of the post-mortem evidence that most implanted stem cells have assumed an astroglial fate (or nestin immunoreactivity) [205,206]. We and others have provided strong evidence that the systemic injection of somatic—and more recently embryonic stem (ES) cell-derived—mouse and human NPCs ameliorates the clinico-pathological features of rodents and non-human primates with experimental autoimmune encephalomyelitis (EAE), the animal model of MS [206,207,208,209,210]. We have demonstrated that this is dependent on multiple mechanisms of action of NSCs within specific microenvironments [211].

In addition to cell replacement [206], we have first described significant neuroprotective and immune modulatory capacities for transplanted (*undifferentiated*) NSCs *in vivo*. It is now established that NSC-mediated bystander actions may take place in the CNS, at the level of the atypical perivascular niches [212], as well as in secondary lymphoid organs [210,213]. Nonetheless, following our own first report that membrane-bound Fas/CD90 ligands (e.g., Apo3L, TRAIL and FasL) were regulating part of the NSC-mediated suppressive effect on encephalitogenic T lymphocytes in the CNS [212], other groups have generated data that describe the mechanisms responsible for these specialized stem cell functions. In general, this has been approached mostly by *in vitro* studies that have utilized immune cell/NSC co-cultures [214]. *In vivo* experiments have provided evidence of short-term persistence of transplanted NSCs into peripheral bodily organs [210,213]. On the other hand, we have also reported the capacity of NSCs to target (and synergize with) immune cells in secondary lymphoid organs following both systemic and subcutaneous (s.c.) NSC injection in EAE mice [210]. Concurrently, the observation of consistent *cellular signalling* between the graft and the host has contributed to challenge the initial idea that stem cell transplants work only via structural cell replacement. Extensive data suggests that, in addition to hierarchical (mother-to-daughter) inheritance and/or segregation of factors regulating cellular identity and/or fate, grafted stem cells communicate and exchange information also horizontally, the different regulators of this latter communication modality including the secretion of growth factors, hormones, cytokines, chemokines and small molecular mediators; cell-to-cell adhesion contacts; exchange of chemical and electrical signals trough gap junctions [215,216]; membranous *nanotubes*, and even the secretion of circular membrane particles and/or EVs. Still, the detailed molecular and cellular mechanism(s) responsible for this multifaceted intercellular communication programme between the stem cell graft and the host remain far from being fully elucidated.

In light of the fact that EVs/exosomes are close copies of the parental cells in terms of their antigenicity (proteins/lipids), profile of miRNAs and mRNAs, but also cytokines and growth factors, the use of exosomes in medicine holds promise. First, EVs overcome many of the limitations of cell-based therapeutics related to safety, manufacturing and availability. Then, studies on the bioavailability of EVs have demonstrated that they are capable of crossing the BBB, which classically acts as a major hurdle in the administration of therapeutic agents targeting the CNS [217,218].

Finally, exosomes have limited immunogenicity compared to live cells, protect their cargoes from degradation, are highly stable in serum and blood, and efficiently deliver their cargo to target cells with reduced off-target effects due to a natural tendency to target specificity [219,220].

### 5.1. Immune Modulatory Potential of Exosomes and EVs

The majority of the early studies that attempted to use EVs or exosomes for therapeutic applications aimed to develop cell-free cancer vaccines based on dendritic-cell derived exosomes (Dex) [221]. Following the observation that Dex released from DC stimulated with tumour-derived antigens mediate tumour rejection through the induction of cytotoxic *T* cells [103], several works have shown that DC-released exosomes have immune stimulatory properties and are able to present antigens and induce Ag-specific MHC II-restricted T cell responses [93,102]. Similarly, it was also shown that after the uptake of tumour-derived exosomes, DCs induce potent CD8^+^ T cell-dependent antitumour effects on syngeneic and allogeneic established mouse tumours, highlighting exosomes as a novel source of tumour-rejection antigens for T cell cross priming [222].

These initial studies led to two phase I clinical trials that investigated the safety of autologous DC-derived exosomes pulsed with tumour peptides for the immunization of stage III/IV melanoma patients and non-small cell lung cancer [223,224]. These studies proved for the first time the feasibility of large-scale production of clinically applicable exosomes and showed their safety in human studies.

At the same time, other works have showed that DC-derived exosomes also have the capacity to induce tolerogenic immune responses in models of graft-versus-host disease [225]; similarly, it was also shown that exosomes from the serum of animals immunized with a specific antigen induce systemic anti-inflammatory and immune-modulatory effects when locally injected in murine models of delayed-type hypersensitivity [95,226].

However, recent results have made it increasingly clear that—in addition to DCs—also stem cells secrete EVs and exosomes with remarkable immune-modulatory capacities [227]. We have recently showed that EVs derived from NSCs have the intrinsic capacity to propagate cellular signalling at a distance, adding additional complexity to the multifaceted mechanisms of interaction that are established between grafted stem cells and the microenvironment [14].

Several recent work suggest that EVs have a beneficial effect in stem-cell based therapies, where they mediate some of the effects of stem cells and promote tissue healing [228]. Bone marrow derived mesenchymal stem cells (MSC) are another well-studied cellular system for exosomes therapeutics. In fact, MSC-derived exosomes induce an (anti-inflammatory) M2-like phenotype in monocytes *in vitro*, leading to polarization of activated CD4^+^ cells to T regulatory cells [229]. Similarly, tumour-derived exosomes have modulatory effects on macrophages that facilitate tumour growth by an escape mechanism from the immune system [230,231]. Also MSC-derived exosomes promote induction and secretion of anti-inflammatory cytokines, resulting in reduced inflammation in cardiac ischemia and renal fibrosis models [232,233,234].

Finally, modified MSC-derived exosomes have promoted CNS regeneration after systemic injection in laboratory animals with experimental cerebral stroke [235].

### 5.2. EVs as a Mediator of Therapeutic Regeneration

Several recent lines of research on the function of EVs point in the direction that they have the intrinsic potential to be exploited as natural delivery vehicles for tissue regeneration. Paracrine signalling via EVs can change the phenotype of target cells through mechanisms that involve the transfer of mRNAs, miRNAs, DNA and/or proteins among others [23,43,236].

Recent data show promising results for the use of MSC- and NSC-derived EVs in various disease models, and importantly for inflammatory disorders of the CNS [237]. At present, cell-based therapeutic strategies for the treatment of stroke are based on the administration of MSCs [238,239,240] and NSCs [241,242,243,244].

Several early studies on the therapeutic potential of MSCs and MSC-derived exosomes focused on myocardial infarction. For example, Lai and colleagues investigated the role of exosomes in a myocardial ischemia/reperfusion model, showing that, human MSC-derived vesicles—of exosomal size and containing exosome-associated proteins—were able to reduce infarct size [245]. Other works have shown that stroke induces significant changes in the MSC expression profile and among the transcripts deregulated following stoke miRNAs are attributed a crucial function in the recovery [246,247,248].

Indeed, it has been suggested that the presence of miRNAs in exosomes secreted by stem cells plays an important role for the fate of recipient cells [249,250,251,252,253].

For example, it was recently shown that miR-133b overexpression in transplanted MSCs results in improved functional recovery, reduced glial scarring and promoted neurite outgrowth, thereby beneficially contributing to the observed effects [235,254]. Similarly, in a zebrafish model of spinal cord injury, miR-133b was shown to be essential for functional recovery, whereas in a model of Parkinson’s disease miR-133b promotes neurite outgrowth [255]. Xin *et al.* [235] demonstrated that miR-133b is down regulated after middle cerebral artery occlusion (MCAo, an animal model of ischemic stroke), while MSC administration resulted in increased miR-133b levels in the ischemic tissue. Furthermore, the ischemic environment induced the up regulation of MSC-derived exosomal miR-133b suggesting a possible role for exosomes in this context [255].

In addition to stroke, the capacity of EVs to mediate therapeutic regeneration has been explored in several other CNS diseases.

For example, in the context of MS a recently published study explored the possibility of exploiting exosomes for therapeutic applications; in fact, it was shown that exosomes released from IFN-γ-stimulated DCs are able to promote myelination and reduce oxidative stress in a lysolecithin-induced demyelination *in vitro* model [256]. Additionally, this work also shown that IFN-γ-stimulated DC-derived exosomes are enriched in miRNA species involved in myelin production and anti-inflammatory response (e.g., miR-219, miR-181a, miR-451, miR-532-5p and miR-665), are directly taken up by ODCs and improve myelination of the motor cortex *in vivo* when applied intranasally in rats. These observations suggest a remarkable therapeutic potential of DC-derived EVs and their possible application in MS therapy [257].

Exosomes also play roles in injury protection and regeneration in several other contexts [258]. For example, exosomes from primary neurons traffic Nedd4-WW (neural precursor cell expressed, developmentally down regulated) domain-binding protein 5 (N4WBP5) [259], which interacts with the ubiquitin ligase Nedd4 and mediates neuroprotection in models of traumatic brain injury [259]. Additionally, it was also shown that ATP [260] induces microglial cells to shed EVs containing the pro-inflammatory cytokine IL-1β [136]. These examples suggest widespread and heterogeneous roles of exosomes in CNS disease and offer the possibility to develop innovative strategies to exploit them to boost their therapeutic potential or target them to hamper their pathogenic roles.

In addition to their inherent modulatory and regenerative capacity, EVs might also be exploited as cell-free drug delivery systems. In fact, EVs purified from cultured cells can be engineered to contain specific molecules of therapeutic relevance [261]. For example, the isolation and systemic administration of DC-derived exosomes expressing TGF-β allows potent inhibition of progress in a mouse model of experimental autoimmune encephalomyelitis [191]. Additionally, the content and functions of EVs can be modulated by changing the culture conditions and/or by giving appropriate stimuli, thus offering an easy and inexpensive way of tailoring the EV content according to the application of interest [262,263,264].

The therapeutic engineering of EVs offers the potential to either specifically modify the EV content or to use EVs as delivery systems for exogenous molecules [218]. By using electroporation of EVs, therapeutic molecules such as siRNA may be incorporated to the vesicles and in the end administered by intravenous injection. This method allowed the brain-specific knockdown of *Beta-secretase* (*BACE*)*1*, a protease with an important role in Alzheimer’s disease, proving *in vivo* the therapeutic potential of EV-based delivery methods [265]. In this study Alvarez-Erviti *et al.* engineered BACE1-siRNA expressing DCs to also express Lysosome-associated membrane protein (Lamp)2 fused to the neuron specific cell penetrating Rabies Virus Glycoprotein (RVG) peptide. By doing so, they were able to deliver the siRNA specifically to neurons, microglia and ODCs, thus achieving a target cell-specific gene knockdown. Following the development of electroporation for RNA loading, several other studies have successfully taken advantage of loading siRNAs and miRNAs into EVs by using transfection agents or vectors [266,267,268].

By overexpressing miR-146b in MSC—a miRNA known to reduce the migration and invasiveness of glioma—MSC-derived exosomes significantly reduced glioma growth [269]. Other than siRNA or miRNA incorporation, controlled loading of protein cargo and expression of targeting ligands on EVs can be achieved [270,271]. For example, loading the anti-inflammatory compound *Curcumin* into EVs led to remarkable protection against LPS-induced inflammation [272].

Moreover, exosome-encapsulated *Curcumin* was shown to inhibit Stat3 in various disease models including LPS-induced inflammation, EAE and glioma [218]. Since the loading efficiency varies among different RNA families and sequences [265], novel techniques need to be developed in order to circumvent side-effects of electroporation for siRNA and miRNA uptake such as aggregate formation or uptake inefficiency. The applications named above underline potential approaches towards tailored EV-mediated regenerative medicine of the CNS.

## 6. Conclusions

The past few years have seen several advances in the field of extracellular vesicles, and it is now well established that EVs/exosomes play an important role in numerous biological processes and pathological conditions. In particular, EVs were shown to be involved in the spreading of toxic protein aggregates in degenerative pathologies of the nervous system, as well as in neuroinflammatory diseases such as MS and stroke.

At the same time, vesicles have attracted great interest for their use as biomarkers, which has recently led to the development of exosome-based commercial diagnostic kits, as well as their use as potential innovative therapeutic tools. The development of cell-free vesicle-based therapeutics is particularly exciting application, because they can recapitulate the modulatory and regenerative potential of cell-based therapies without the intrinsic safety concerns connected to the administration of live cells; in particular, the capacity of EVs to cross the BBB makes them particularly suitable for the treatment of CNS diseases.

Additionally, the potential of vesicles as therapeutic agents has been further exploited by engineering EV surface molecules in order to target them to specific organs or cell types. This is a particularly exciting development, as it will allow the targeted delivery of small molecules, proteins or nucleic acid in a safe, efficient and specific way. To better exploit exosomes for therapeutic purposes numerous group are trying to characterize the molecular machinery responsible for sorting the protein and RNA cargos towards vesicles. Despite we are still far from a precise understanding of this process, it is now getting clear that—at least in some cases—short RNA motifs can be recognized by specific carrier proteins which—in turn—shuttle them towards exosomes. However the picture is more complicated, because different motifs have been identified in different contexts and different mechanisms of sorting have been proposed. Whether they are alternative mechanisms that operate in different species and/or cell types or whether they all co-exist and co-operate is a question that will only be answered with new experiments and innovative approaches.

A better understanding of all aspects of exosomes biology has the potential to lead to the development of innovative and successful high clinical impact therapeutics for neurodegenerative disorders. For further information on the basic properties of EVs, their involvement in various diseases, their role in cell-cell communication, as drug delivery vehicles, *etc.*, the reader is referred to the various reviews in this focus edition [19,156,273,274,275].
